# *Trypanosoma cruzi* genetic diversity associates with serological diagnostic assay performance

**DOI:** 10.21203/rs.3.rs-10119431/v1

**Published:** 2026-07-13

**Authors:** Julie Thompson, Eric Dumonteil, Claudia Herrera

**Affiliations:** Department of Tropical Medicine and Infectious Disease, Vector-Borne and Infectious Disease Research Center, Celia Scott Weatherhead School of Public Health and Tropical Medicine, Tulane University, New Orleans, LA, USA.

**Keywords:** Chagas disease, diagnosis, antibody, genotype, parasite

## Abstract

**Background:**

Infections with *Trypanosoma cruzi* can lead to Chagas disease. Diagnosis principally relies on serological assays, although there is no gold standard assay, and discordant results among tests are increasingly reported. Parasite diversity has been proposed as a potential factor underlying variable diagnostic performance, but clear associations have not been detected yet. Therefore, we tested here how parasite strain composition in infected hosts - the “cruziome”- may associate with serological outcomes in this cohort.

**Methods:**

Archived blood samples from patients from Argentina, Honduras and Mexico were stratified as confirmed positive or serodiscordant based on previous serological testing, and *T. cruzi* parasites were successfully genotyped in 73 patients.

**Results:**

A total of 532 mini-exon sequences were obtained (7.3 ± 0.5 haplotypes/individual), covering TcI, TcII, TcIV, TcV and TcVI DTUs, in similar proportions among countries, but variable proportions among individuals. Phylogenetic analysis indicated a significant geographic structuring of TcI, TcII, TcV and TcVI among countries, as well as clustering associated with discordant serology for TcI, TcII and TcV DTUs. Between 5–12 SNPs among these DTUs supported the association with discordant serology, and their frequency among countries mirrored that of serodiscordance.

**Conclusion:**

These results point to the cruziome and genetic differentiation within TcI, TcII and TcV DTUs to explain a large part of geographic differences in diagnostic performance. Thus, it is critical to account for the local diversity of *T. cruzi* strains throughout the continent for the development of improved diagnosis.

## Introduction

Infections with *Trypanosoma cruzi*, a protozoan parasite, can lead to Chagas disease, a major health problem in the Americas. Indeed, there are an estimated 10 million infected persons worldwide [1], of whom 30–40% may progress to severe clinical disease characterized by cardiac and/or digestive manifestations [2]. Diagnosis relies primarily on serological assays detecting antibodies against the parasite. However, there is no gold standard assay. Thus, current recommendations require two distinct assays for the confirmation of cases, and when discordant, a third assay needs to be performed [3]. This testing algorithm is lengthy and complicates case identification, leading to important underdiagnosis of infections [4–8].

Critically, serodiscordance is increasingly reported, particularly with samples from Central and North America [8–12] and it poses a challenge for accurate diagnosis as cases are often dismissed as false positives given their apparent non-consistent reactivity across assays. Some studies suggested that serodiscordance may be associated with low level antibodies lingering after a cured infection [13]. However, our previous work indicated that parasite burden was identical between serodiscordant and confirmed seropositive samples [10]. On the other hand, differences in antibody levels against diagnostic antigens suggested that serodiscordance reflected a distinct humoral response, possibly attributed to the infecting parasite strains or host factors [10]. Indeed, *T. cruzi* parasites are classified into seven major lineages, TcI to TcVI and TcBat referred to as discrete typing units (DTUs). The initial description of their geographic distribution suggested that TcI was the predominant DTU circulating in the northern hemisphere, while non-TcI (TcII, TcV and TcVI) predominated in the southern hemisphere [14,15]. Thus, difference in circulating DTUs were also thought to contribute to serodiscordance. However, new evidence over the past decade clearly shows that the geographic distribution of DTUs is more complex, with TcII, TcV and TcVI increasingly detected in Central America, Mexico and the US [16–27]. Furthermore, these studies and others [28] also highlighted that many infections were composed of multiple parasite strains, including from different DTUs. This multiplicity of infection can have important implications for the host immune response and disease progression [29,30] and we proposed the term “cruziome” to describe the parasite assemblage of strains infecting a host [31].

Previous efforts to associate parasite DTUs and genetic diversity with serodiscordance suggested a possible association, but the evidence remained weak due to a small sample size and limited phylogenetic resolution to assess *T. cruzi* genetic diversity and the complete cruziome [17]. In dogs, serodiscordance among tests is also an issue [32,33], and a previous analysis similarly suggested that TcI genetic diversity could be associated with discordant serology [20]. Based on the above, we tested here for the potential role of *T. cruzi* strain diversity on diagnostic test accuracy by performing high resolution parasite genotyping based on next generation sequencing for optimal resolution of the cruziome, applied to a large cohort of patient samples from Argentina, Honduras and Mexico. We examined how different aspects of the cruziome composition may associate with serological outcomes in this cohort.

## Materials and methods

### Ethics Approval and Consent to participate

The present study was approved by Tulane University Institutional Review Board (No. 2018–2237) and used de-identified archived samples from a previous study on congenital *T. cruzi* transmission and collected following written informed consent.

### Patient samples

Samples consisted in maternal blood/DNA samples collected at birth in Argentina (N=160), Honduras (N=233) and Mexico (N=110) for a total of 503 samples [34]. T. cruzi parasites were successfully genotyped in a subset of maternal samples (see below). Metadata associated with these samples included extensive serological testing for *T. cruzi* infection based on Stat-Pak (Chembio Diagnostics) and T-detect (InBios) rapid tests and a confirmatory recombinant ELISA (Wiener), as well as *T. cruzi* PCR and parasite burden measured by qPCR as detailed before [34]. Serological and PCR data were also available from their matching umbilical cord blood samples. The samples were stratified as confirmed positive when all three serological assays (two rapid tests and one ELISA) were reactive and concordant, and serodiscordant when the ELISA was negative while zero, one or two rapid tests may be reactive.

### *T. cruzi* genotyping

Parasite genotyping was performed by amplifying the mini-exon marker using a multiplex PCR as described by Souto et al., which generates products of different sizes according to the DTU [35]. In parallel, an alternative primer set amplifying a larger fragment of 500 bp of the mini-exon gene was used as previously described. [36]. PCR products from both reactions were mixed, purified and processed for sequencing on an Illumina MiSeq platform. Raw FASTQ reads were demultiplexed for each sample and competitively mapped to reference sequences of the mini-exon covering all seven DTUs in Geneious Prime, and SNPs were called from each individual DTU present using FreeBayes or Geneious SNP utility. Haplotypes from each DTU were reconstructed based on these SNPs, and their relative abundance was estimated based on the proportion of reads covering each haplotype. Only sequences present at a frequency >1% of reads were considered for further analysis. These haplotype sequences were aligned using MAFFT [37], and phylogenetic analysis was performed in FastTree 2, which infers approximately-maximum-likelihood phylogenetic trees from alignments based on a generalized time-reversible (GTR) model of nucleotide evolution [38]. Mini-exon reference sequences from all DTUs were included in the trees to identify the respective DTU clusters (TcI: H1, EF576846; SilvioX10, CP015667; TcII: Tu18, AY367125; TcIII: M5631, AY367126; TcIV: 92122102r, AY367124; TcV: SC43; AY367127, TcVI: CL, U57984, and TcBat: TCC949cl3, KT305873). Trees were visualized and formatted with FigTree. A total of 73 DNA samples from maternal blood were successfully genotyped, including samples from Argentina (N=20), Honduras (N=24) and Mexico (N=29) from which we obtained their detailed cruziome DTU and haplotype composition. Sequences have been deposited into NCBI Genbank database under accession number PZ560951–PZ561482.

### Data analysis

The samples were classified as confirmed positive (N=44) or serodiscordant (N=29). Of these discordant samples, 3 (10%) were negative in all serological assays, 22 (76%) were reactive in a single rapid test, and 4 (14%) were reactive with 2 rapid tests. We then tested for potential differences in their cruziome by considering its diversity at multiple levels. First, we tested for differences in overall cruziome complexity/size by comparing the average number of *T. cruzi* DTUs and sequence haplotypes infecting individuals (alpha diversity) with Student t tests.

Second, we tested for an association with the presence/absence of specific parasite DTUs based on X^2^ tests, and with the average composition of the cruziome (beta diversity). This was tested by one-way permutation ANOVA (PERMANOVA) with 10,000 permutations. Differences in cruziome composition among countries were also assessed by linear discriminant analysis (LDA).

Third, we assessed the potential contribution of individual sequence haplotypes by performing phylogenetic analysis of *T. cruzi* sequences from each DTU. Sequences were aligned using MAFFT and trees generated with FastTree as above with each DTU analyzed separately. The statistical significance of sequence clustering according to country of origin and with diagnostic discordance was assessed by one-way PERMANOVA based on Tamura-Nei genetic distances among sequences as implemented in Past 4 [39] and linear discriminant analysis (LDA) was also performed.

Finally, we identified SNPs in the sequence alignments of the mini-exon sequences from each DTU, which were numbered based on SNP position within reference sequences (H1cl2, EF576846.1 for TcI; Tu18, AY367125.1 for TcII and SC43, AY367127.1 for TcV). SNPs were tested for their association with diagnostic outcome by comparing SNP frequencies in confirmed positive and serodiscordant samples with X^2^ tests and odds ratios were calculated. Bonferroni correction was applied to account for multiple testing of SNPs. Multivariate logistic regression models were then built to integrate multiple SNPs from the respective DTUs as predictors of diagnostic performance. Akaike information criteria (AIC) was used to rank the models and only the best models were retained. The association of SNPs with the performance of individual serological assays was also tested in the same manner for Stat-Pak and T-detect rapid tests and for Wiener ELISA. Finally, SNP frequencies were also calculated among the different countries and compared by X^2^ tests.

## Results

### Serodiscordance is a stable characteristic of plasma samples

**Figure 1.**
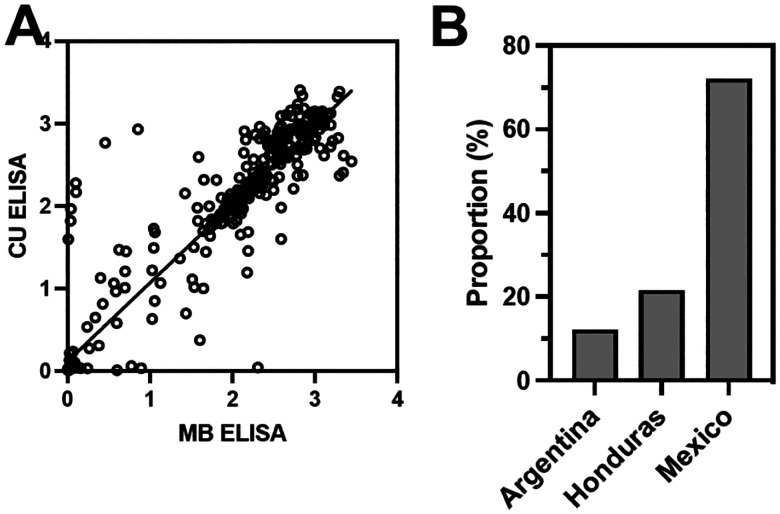
Serodiagnostic characterisitics of samples. (**A**) Correspondence of ELISA optical density readings between maternal blood (MB) and umbilical cord blood (UC). IgG levels of umbilical cord blood samples matched well to those in maternal blood (R^2^=0.90, P<0.0001). Maternal blood samples with discordant serology were also discordant in their corresponding cord blood (Kappa=0.95, P<0.0001). (**B**) Proportion of discordant serology among PCR positive samples by country.

We first characterized the serodiscordance of our panel of maternal plasma samples by testing its stability and reproducibility by comparing these samples with their matching umbilical cord plasma samples, which are considered to mostly contain maternal antibodies [40,41]. Comparison of anti-*T. cruzi* IgG levels measured by ELISA between maternal and cord blood indicated a nearly perfect correlation (Figure 1A, R^2^=0.90, P<0.0001), and serodiscordance was similar for maternal samples and corresponding cord blood samples (Kappa index=0.95, P<0.0001). This strongly supported the presence of a distinct antibody profile among these samples, as suggested before [10]. Also, as reported before [10,34], the proportion of serodiscordance was highest in samples from Mexico, intermediate in samples from Honduras, and lower in samples from Argentina (Figure 1B).

### Genotyping of *T. cruzi* reveals extensive genetic diversity and frequent mixed infections

**Figure 2.**
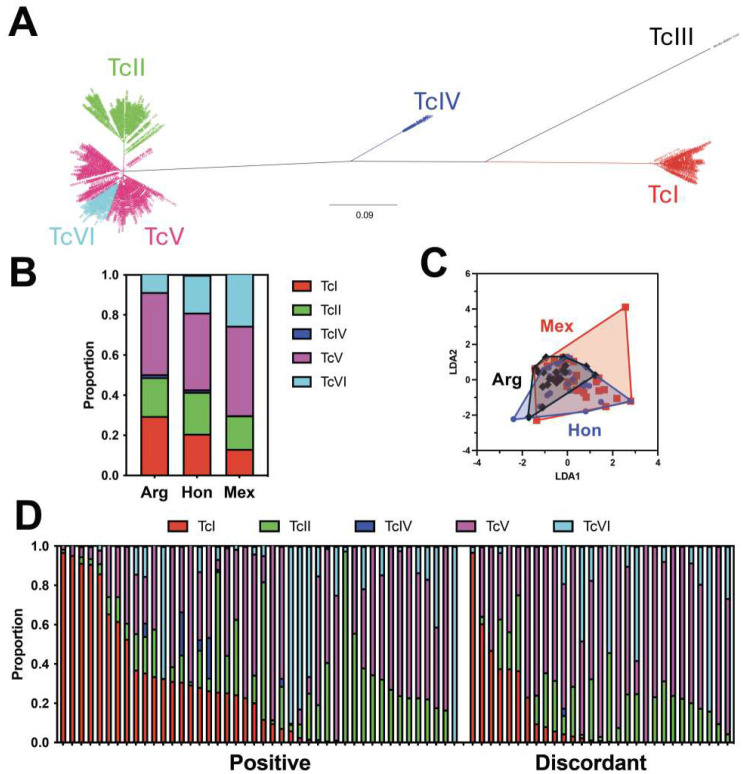
Genotyping of *T. cruzi* in maternal blood. (**A**) Phylogenetic analysis of 532 *T. cruzi* mini-exon sequences recovered from maternal blood, corresponding to an average of 7.3 ± 0.5 haplotypes per individual, indicating the presence of TcI, TcII, TcIV, TcV and TcVI parasite DTUs. (**B**) Average cruziome composition by country and (**C**) LDA of cruziome composition by country, indicating some minor variability in composition that did not reach statistical significance (PERMANOVA F=1.87, P=0.10). Parasite DTUs are color-coded as indicated. (**D**) Cruziome composition among individual subjects stratified as confirmed positive or discordant for *T. cruzi* serology. Each column represents a subject and parasite DTUs are color-coded as indicated. The large majority of individuals presented infections with multiple parasite DTUs in variable proportions.

The sequencing of *T. cruzi* mini-exon amplicons from 73 maternal blood samples generated a total of 532 sequences, corresponding to an average of 7.3 ± 0.5 haplotypes per individual. Phylogenetic analysis indicated that these sequences clustered with TcI, TcII, TcIV, TcV and TcVI reference sequences, highlighting the extensive diversity of DTUs infecting these subjects. The average cruziome included multiple DTUs in variable proportions across countries, but this did not reach statistical significance (Figure 2B and C; PERMANOVA F=1.87, P=0.10), suggesting broadly comparable geographic distribution of DTUs among this sample of infected mothers from these countries. Analysis of individual cruziomes also highlighted that the large majority of infections included multiple DTUs in variable proportions (72/73 (98.6%) had multiple DTUs present) (Figure 2D). Individual cruziomes could then be stratified according to *T. cruzi* serological testing outcome.

### Cruziome complexity/size does not associate with serological discordance

**Figure 3.**
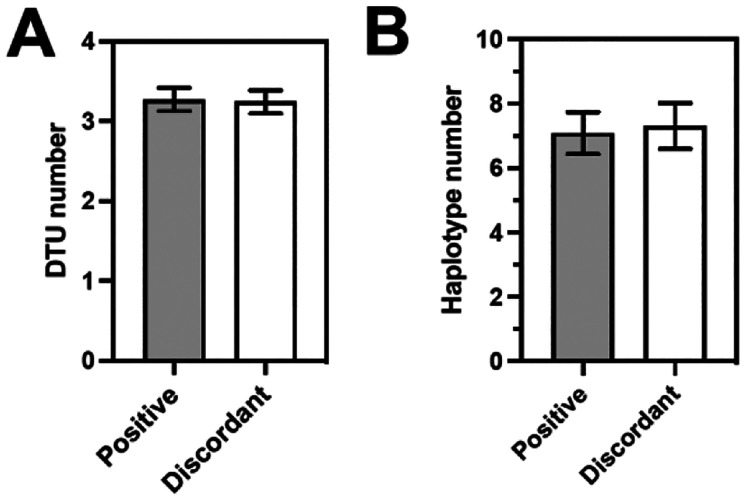
Role of cruziome complexity/size. Cruziome complexity/size was measured as the number of *T. cruzi* DTUs (**A**) or number of haplotypes (**B**) per individual. Positive and discordant samples presented an identical level of cruziome complexity/size (t test, P=0.88 and P=0.82 for DTUs and haplotypes, respectively). The effect of cruziome complexity on maternal blood parasite burden indicated that parasite burden was significantly associated with the number of infecting DTUs.

We explored how the cruziome composition and diversity may associate with serological diagnostic discordance by considering different levels of diversity. First, we tested for potential differences in cruziome complexity/size and compared the number of parasite DTUs and sequence haplotypes infecting individuals (Figure 3A and B), but neither the number of DTUs nor the number of haplotypes were associated with serodiscordance (*t* test, P=0.88 and P=0.82 for DTUs and haplotype number, respectively).

### Unique *T. cruzi* sequence haplotypes associate with serological discordance.

**Figure 4.**
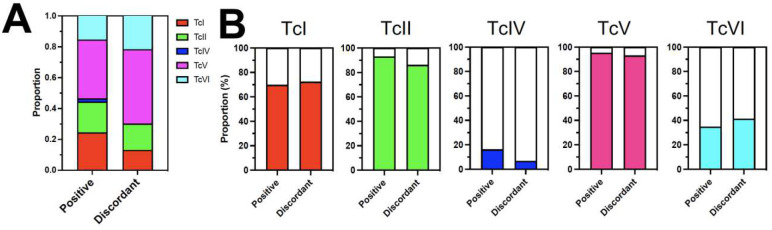
Role of DTU composition and presence. Cruziome DTU composition (**A**) and the presence/absence of each DTU (**B**) were compared between positive and discordant samples. DTU composition tended to show some difference between positive and discordant samples, but this did not reach statistical significance (PERMANOVA, F=1.99, P=0.12). Each DTU was equally present in positive and discordant samples (X^2^, d.f.=1, P=0.81, P=0.32, P=0.22, P=0.68 and P=0.57 for TcI, TcII, TcIV, TcV and TcVI, respectively).

Next, we tested for differences in cruziome composition based on the proportion of the different DTUs (Figure 4A) or the presence/absence of specific DTUs (Figure 4B) associated with discordant serology. None of these variables presented significant differences between confirmed positive and serodiscordant samples (PERMANOVA, F=1.99, P=0.12 for overall composition, and X^2^, d.f.=1, P=0.81, P=0.32, P=0.22, P=0.68 and P=0.57 for TcI, TcII, TcIV, TcV and TcVI, respectively).

**Figure 5.**
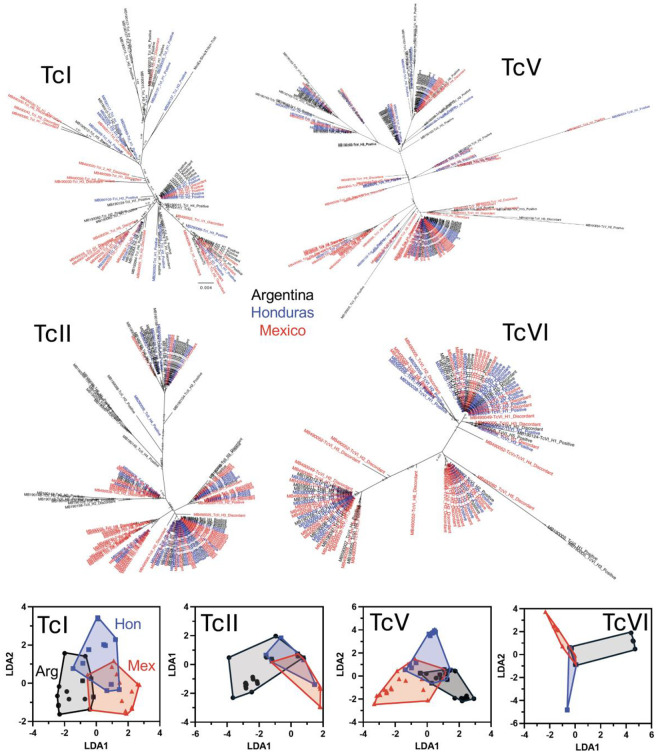
Within DTU parasite diversity by country. Phylogenetic analysis of *T. cruzi* haplotypes within DTUs indicates significant geographic structuring among each DTU tested, with a significant clustering of sequences by country (PERMANOVA, F=3.03, P=0.039; F=5.99, P=0.008; F=9.29, P=0.0001; and F=61.7, P=0.034 for TcI, TcII, TcV and TcVI, respectively).

Lastly, we examined the potential contribution of individual haplotype sequences, which may reflect the presence of genetically different parasite strains. Phylogenetic analysis of mini-exon sequences from TcI, TcII, TcV and TcVI DTUs indicated extensive sequence diversity within DTUs (Figure 5), as observed before [42–44]. Accordingly, this diversity reflected a geographic structuring that reached statistical significance for TcI, TcII, TcV and TcVI (PERMANOVA F=3.03, P=0.039; F=5.99, P=0.008; F=9.29, P=0.0001 and F=61.73, P=0.034, for TcI, TcII, TcV and TcVI, respectively), indicating a significant level of genetic clustering among parasite sequences according to the country of origin within each DTU (Figure 5).

**Figure 6.**
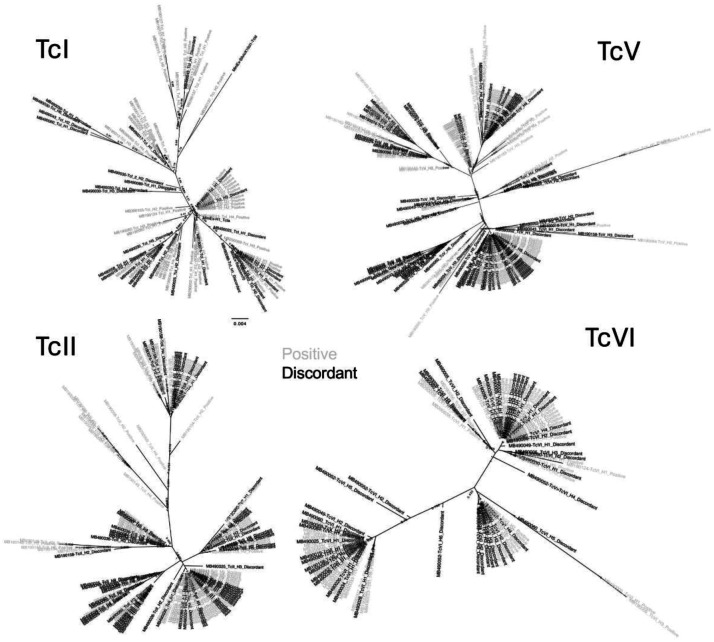
Association of haplotype sequence diversity with diagnostic performance. Phylogenetic analysis of *T. cruzi* haplotypes within DTUs is shown. Sequence names are color-coded depending on their association with a confirmed positive or discordant serology. There was a significant clustering of sequences from individuals with discordant serology for TcI, TcII and TcV DTUs (PERMANOVA F=3.83, P=0.023; F=4.73, P=0.034 and F=7.24, P=0.002 for TcI, TcII and TcV, respectively), while this association was not detected for TcVI (F=3.231, P=0.183).

**Figure 7.**
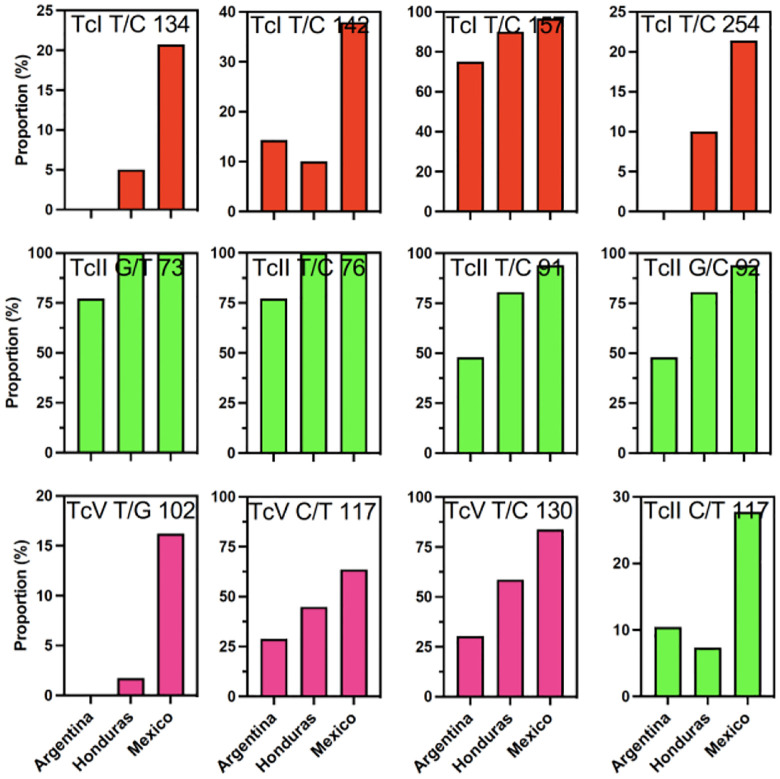
SNP and discordant serology frequency among countries. The frequency of the indicated *T. cruzi* mini-exon SNPs associated with serological discordance was examined among countries. All SNPs presented significant differences in frequency among countries (d.f.=2, X^2^=9.4, P=0.009 for TcI T/C 134; X^2^=6.8, P=0.033 for TcI T/C 142; X^2^=6.3, P=0.043 for TcI T/C 157; X^2^=9.1, P=0.011 for TcI T/C 254; X^2^=9.6, P=0.008 for TcII C/T 117; X^2^=27.6, P<0.0001 for TcII G/T 73; X^2^=27.6, P<0.0001 for TcII G/T 76; X^2^=32.8, P<0.0001 for TcII C/T 91; X^2^=32.8, P<0.0001 for TcII G/C 92; X^2^=22.2, P<0.0001 for TcV T/G 102; X^2^=17.4, P=0.0002 for TcV T/C 117; X^2^=43.4 P<0.0001 for TcV T/C130), and SNP frequencies followed a similar pattern as that of discordant serology.

**Figure 8.**
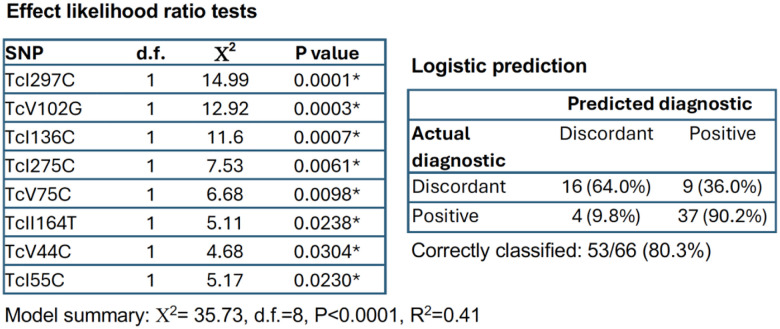
Logistic model predicting diagnostic outcome based on parasite SNPs. Seven SNPs from TcI, TcII and TcV DTUs were sufficient to explain diagnostic outcome (P<0.0001, R^2^=0.41) with high accuracy as 80.3% of samples were correctly identified as serodiscordant or confirmed positive based on this combination of SNPs.

Remarkably, sequences derived from serodiscordant samples were also over- or under-represented in some tree branches for TcI, TcII and TcV, and this phylogenetic clustering was statistically significant (PERMANOVA F=3.83, P=0.023; F=4.73, P=0.034 and F=7.24, P=0.002 for TcI, TcII and TcV, respectively)(Figure 6). Such an association was not detected for TcVI (F=3.231, P=0.183). These data strongly suggested that strain genetic diversity within DTUs is associated with serodiscordance for at least TcI, TcII and TcV DTUs. To explore this association in more detail, we examined SNPs within the mini-exon sequences, and identified several SNPs in TcI, TcII and TcV sequences which were associated with discordant serology, and the statistical significance of several of these SNPs remained after Bonferroni correction, which is highly conservative (Table 1). Furthermore, the frequency of these SNPs varied significantly among the three countries (Figure 7), mirroring the observed frequency of serodiscordance. Lastly, we built a multivariate logistic regression model which explained diagnostic outcome (confirmed positive or serodiscordant) with high accuracy (80% correct prediction, P<0.0001) based on a small number of SNPs from TcI, TcII and TcV parasites (Figure 8). Similar models were built to assess the contribution of parasite SNPs on the outcome of individual assays, and SNPs subsets were found to be major factors driving the outcome of all assays (Supplementary Figure 1; R^2^=0.37, R^2^=0.27 and R^2^=0.26 for Stat-Pak, T-Detect, and Wiener ELISA, respectively). Thus, 75–88% of diagnostic outcomes could be correctly explained by *T. cruzi* SNPs combinations. Together, these results strongly supported the circulation of genetically diverse *T. cruzi* strains from TcI, TcII and TcV DTUs within the three countries, with some of these strains significantly associated with discordant serology.

## Discussion

The complex diagnostic algorithm for *T. cruzi* infections is a major limitation for the rapid identification of cases and accurate disease surveillance, and discordance among serological assays has been a lingering problem [8–12], particularly relevant in Central America and Mexico. Understanding the causes of serodiscordance is critical to guide the development of improved diagnostic tests with better performance, and previous work suggested that *T. cruzi* strain diversity could be a contributing factor. Indeed, a complex geographic distribution of parasite strains and DTUs is emerging, adding to the observation that antigens used in diagnostic tests may not be as conserved as initially thought [42]. Furthermore, the multiple strains and DTUs composing the cruziome infecting hosts may also affect immune response and diagnostic performance, acting as an additional confounder. Thus, we examined here the potential association between serodiscordance and cruziome composition at multiple levels following the detailed genotyping of parasites from a large cohort of infected patients.

A first major observation was that most (>98%) of patients were infected with multiple DTUs, which overall distribution was highly similar among patients from Argentina, Honduras and Mexico. This largely confirms our previous genotyping effort on some of these samples with a less sensitive method [17,34], strengthening the idea that TcII, TcV and TcVI are important DTUs circulating in Central America and Mexico, while TcI is also highly relevant for Argentina. Together with many other studies [16–26], these results indicate that the DTU distribution in the Americas initially proposed [14,15] needs to be revised and better defined. In addition, we also detected a significant geographic structuring for TcI, TcII, TcV and TcVI sequences, suggesting some level of local differentiation within these DTUs. Geographic structuring of *T. cruzi* across the Americas has been described before within TcI [42,43,45–47], as well as within TcIII and TcIV which have been divided into northern and southern clades [48,49]. TcII also seems to present some level of geographic variability within Brazil [50]. Thus, our observations expand these findings and suggest that most if not all *T. cruzi* DTUs are geographically structured. This has important implications as biological properties of parasite strains may also vary accordingly, leading to potential differences in clinical disease progression or response to treatments. For example, geographical variations in clinical outcomes in the BENEFIT trial have been observed [51], stressing the need to better understand *T. cruzi* diversity throughout the continent to account for this heterogeneity.

Our data also suggest that most infections involve multiple *T. cruzi* strains/DTUs, strengthening the concept of cruziome and its relevance to understand host response to infection, with implications ranging from diagnosis to pathogenesis and treatment [31]. Regarding the role of cruziome composition in affecting serological results, we found that serodiscordance was not associated with cruziome complexity/size, overall DTU composition, or the presence/absence of any specific DTU. Rather, we detected an association with unique mini-exon sequence haplotypes for TcI, TcII and TcV, which differed from haplotypes from these DTUs associated with a confirmed positive diagnosis. This was further strengthened by the identification of several SNPs within these sequences that were significantly associated with serodiscordance, and which frequency followed a matching geographic distribution. Indeed, SNP variants associated with serodiscordance were detected at lower frequencies in Argentina and higher frequencies in Mexico, and seven SNPs from TcI, TcII and TcV were sufficient to accurately predict >80% of serological diagnostic outcomes. Remarkably, this pattern was consistent across serological diagnostic assays as their individual performance was similarly well predicted by parasite SNP combinations.

Together with initial observations in humans [17] and dogs [20], these results provide strong evidence for the major contribution of *T. cruzi* parasite genetic diversity to serodiscordance. While SNPs within the mini-exon marker are of limited usefulness to infer specific biological differences, these likely reflect broader genomic differentiation of parasites within DTUs as observed before [42,44]. Thus, we may hypothesize that infections with some *T. cruzi* strains within these DTUs may lead to antibody repertoires targeting parasite antigens different than those included in current diagnostic assays, leading to serodiscordance. Indeed, these assays rely mostly on antigens derived from strains from the southern cone, which may not reflect strains from the northern hemisphere [42,52]. A growing number of observations indicating that epitope profiles recognized by antibodies from *T. cruzi* infected patients are mostly private, i.e. unique to individuals, with very few shared epitopes recognized by antibodies from multiple patients [42,53] further support this idea. Analysis of antibody repertoires detected in *T. cruzi* infected dogs and macaques also indicate mostly private repertoires [54,55].

## Conclusion

In conclusion, the analysis of the cruziome of a large cohort of *T. cruzi* infected patients confirmed the strong predominance of infections with multiple parasite strains/DTUs, with a comparable DTU composition among patients from Argentina, Honduras and Mexico. Furthermore, significant geographic structuring was detected within TcI, TcII, TcV and TcVI, confirming and expanding earlier observations, which has important implications for diagnosis. Remarkably, a significant association between SNPs within TcI, TcII and TcV with serodiscordance was detected and seven SNPs were sufficient to explain most of the geographic differences in diagnostic performance. These results imply that it is critical to consider the local diversity of *T. cruzi* DTUs throughout the continent for the development of improved diagnosis with optimal performance.

## Supplementary Material

Supplementary Files

This is a list of supplementary files associated with this preprint. Click to download.

- SupplFig1.pdf

## Figures and Tables

**Table 1.** SNPs associated with serodiscordance

DTU	SNP ID^#^	Positive	Discordant	P value*	Odds Ratio	OR 95%CI
TcI	T/C 279	C, 1/54 (1.8%)	C, 6/31 (19.3%)	0.005*	12.72	1.45–111.38
TcI	C/T 287	T, 7/54 (13.0%)	T, 11/31 (35.5%)	0.016	3.69	1.25–10.90
TcI	T/C 302	C, 9/54 (16.7%)	C, 1/31 (3.2%)	0.043	0.16	0.02–1.38
TcI	T/C 398	C, 2/54 (3.7%)	C, 6/30 (20.0%)	0.016	6.50	1.22–34.59
TcII	G/T 297	G, 77/87 (88.5%)	G, 65/66 (98.5%)	0.010	8.44	1.05–67.70
TcII	C/T 300	C, 77/87 (88.5%)	C, 65/66 (98.5%)	0.010	8.44	1.05–67.70
TcII	C/T 314	C, 59/87 (67.8%)	C, 57/66 (86.4%)	0.007*	3.00	1.30–6.92
TcII	G/C 315	G, 59/87 (67.8%)	G, 57/66 (86.4%)	0.007*	3.00	1.30–6.92
TcII	C/T 340	T, 8/87 (9.2%)	T, 18/66 (27.3%)	0.003*	3.70	1.49–9.17
TcV	T/G 329	G, 1/109 (0.9%)	G, 13/75 (17.3%)	<0.0001*	22.64	2.89–177.28
TcV	C/T 344	T, 45/109 (41.3%)	T, 44/75 (58.7%)	0.020	2.02	1.11–3.66
TcV	T/C 356	C, 53/109 (48.6%)	C, 54/75 (72.0%)	0.001*	2.72	1.45–5.09

# numbers in SNP ID refer to nucleotide position in reference mini-exon sequences.

* indicate P values that remained statistically significant after Bonferroni correction.

## Data Availability

Sequences have been deposited into NCBI Genbank database under accession number PZ560951–PZ561482.
